# Large-Scale Population Study of Human Cell Lines Indicates that Dosage Compensation Is Virtually Complete

**DOI:** 10.1371/journal.pgen.0040009

**Published:** 2008-01-18

**Authors:** Colette M Johnston, Frances L Lovell, Daniel A Leongamornlert, Barbara E Stranger, Emmanouil T Dermitzakis, Mark T Ross

**Affiliations:** 1 X Chromosome Group, Wellcome Trust Sanger Institute, Wellcome Trust Genome Campus, Hinxton, Cambridge, United Kingdom; 2 Population and Comparative Genomics Group, Wellcome Trust Sanger Institute, Wellcome Trust Genome Campus, Hinxton, Cambridge, United Kingdom; Massachusetts General Hospital, United States of America

## Abstract

X chromosome inactivation in female mammals results in dosage compensation of X-linked gene products between the sexes. In humans there is evidence that a substantial proportion of genes escape from silencing. We have carried out a large-scale analysis of gene expression in lymphoblastoid cell lines from four human populations to determine the extent to which escape from X chromosome inactivation disrupts dosage compensation. We conclude that dosage compensation is virtually complete. Overall expression from the X chromosome is only slightly higher in females and can largely be accounted for by elevated female expression of approximately 5% of X-linked genes. We suggest that the potential contribution of escape from X chromosome inactivation to phenotypic differences between the sexes is more limited than previously believed.

## Author Summary

The males and females of many species are distinguished by their inheritance of different sets of sex chromosomes. This creates a significant imbalance in gene number between the sexes. Dosage compensation is the correction for this imbalance and is achieved by regulating gene activity across entire sex chromosomes. For example, human females have two X chromosomes and males have only one. Dosage compensation in humans involves X chromosome inactivation, which is the silencing of one X chromosome in female cells. Some genes are known to escape the silencing process and so are expressed at higher levels in females than males. We have investigated the extent to which such genes disrupt dosage compensation by comparing the activity of X chromosome genes in a large number of human male and female cell lines. We have shown that gene expression from the X chromosome pair in female cell lines is only slightly higher than from the single X in males. The small difference can be accounted for by increased female expression of approximately 5% of X chromosome genes. We conclude therefore that dosage compensation in these human cell lines is virtually complete, and we suggest that differences in X chromosome gene expression between males and females may be less extensive than previously thought.

## Introduction

Dosage compensation is a regulatory process that alters gene expression along entire X chromosomes resulting in equivalent levels of X-linked gene products in males and females. Dosage compensation has evolved independently several times and is achieved in various ways. In Drosophila melanogaster, for example, the male X chromosome is hypertranscribed, doubling the output of X-linked genes (reviewed in [1] and [2]). This balances gene expression between the sexes and also satisfies the second requirement of a dosage compensation system, which is to balance X chromosome gene expression with that of the autosomes. The situation in mammals is more complex. Inactivation of one of the X chromosomes in female mammals [3] balances X-linked gene expression between males and females. However, if this were the only component of the mammalian system, the single active X chromosome of females and males would effectively make both sexes aneuploid with respect to autosome gene expression. Ohno hypothesized therefore that balance would be achieved by doubling the output from the male X (and active female X) [4]. The hypothesis was confirmed recently when 2-fold upregulation of the X chromosome was demonstrated for both human [5] and mouse [6].

The molecular mechanism for this X chromosome upregulation in mammals is unknown. By contrast X chromosome inactivation (XCI) has been known about for almost fifty years and is extensively characterized. XCI is a complex and tightly regulated process unique to mammals that results in heterochromatization and transcriptional silencing of one of the female X chromosomes (for a review see reference [7]). In eutherian mammals, the maternal or paternal X chromosome is inactivated randomly early in embryogenesis, and once established the pattern is mitotically stable. XCI was first suggested in 1961 to explain mosaic phenotypes seen in female mice heterozygous for sex-linked mutations in coat colour genes [3]. The theory was supported by the observation that cloned fibroblasts from human females heterozygous for an electrophoretic protein variant from the X-linked gene *G6PD* expressed either the paternal or the maternal allele, but not both [8]. Similar observations were reported for *HPRT* [9], *PGK* [10,11] and a number of other X-linked genes.

This pattern of complete silencing of one allele in females is seen for the majority of X-linked genes tested. However, the finding that the steroid sulphatase gene (*STS*) was always expressed in female fibroblast clones with one *STS*-deficient allele, regardless of which X was inactivated, suggested that some genes are not subject to XCI [12]. This “escape” from XCI results in differential expression of *STS* loci on the active X (X_a_) and inactive X (X_i_) chromosomes: clones expressing *STS* from the X_i_ have approximately half the level of STS enzyme activity of clones expressing from the X_a_ [13].

The use of rodent-human hybrid cell lines retaining an inactive human X chromosome has contributed greatly to our knowledge of escape from X inactivation [14–16]. This approach has the advantage of being able to assay gene expression from the X_i_ without the interference of the active copy. The largest study of this type has estimated that minimally 16% of human genes escape from XCI [16]. In a complementary approach, the same authors compared expression from maternal and paternal alleles of 94 genes in a panel of cell lines with skewed XCI and found that 15% of these were consistently expressed from both X chromosomes [16].

These approaches can detect low levels of expression from X_i_. However, measuring the effect on dosage compensation requires a method to compare gene expression between the sexes. Expression microarrays, designed using the annotated X chromosome sequence [17], are suitable for such comparisons. Microarrays have previously been used to identify human genes escaping XCI by comparing gene expression in cell lines with supernumerary X chromosomes [18], in male and female lymphocytes [19], and in a range of male and female tissues [20]. A genome-wide survey of sex-differences in gene expression in lymphoblastoid cells also yielded several examples of X chromosome genes with elevated female expression levels [21].

In the light of this reported widespread escape from X inactivation, we sought to determine its effect on dosage compensation in the largest comparison to date of X chromosome gene expression in females and males. Here we report analysis of a microarray expression dataset obtained using lymphoblastoid cell lines from 210 individuals in four populations. We show that the proportion of X-linked genes with significantly higher expression in females is around 5%, and that dosage compensation in these cell lines is virtually complete.

## Results

### Expression from the X Chromosome in Male Cell Lines Is Upregulated 2-Fold

Microarray data from the Gene Expression Variation project (GENEVAR [22–24]) were analysed for 210 unrelated individuals from the four HapMap populations [25], designated CEU, CHB, YRI and JPT (see Methods).

**Figure 1:**
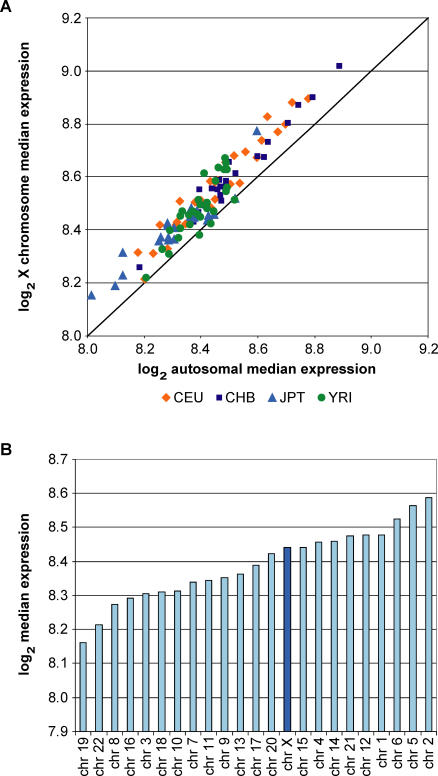
Gene Expression from the Single Male X Chromosome Is Similar to Expression from Autosome Pairs (A) Scatter graph of median expression of 11,952 autosomal genes against median expression of 371 X chromosome genes for 105 males. The diagonal indicates the position at which median expression levels from X and autosomes are the same. (B) Median expression for each autosome pair and for X. Medians were calculated for each gene for 30 YRI males, and then medians were calculated for each autosome and for X. Data are ranked in order of increasing median expression.

First, we identified 371 genes on the X chromosome and 11,952 genes on autosomes that are expressed in the cell lines (see Methods). We then compared gene expression from autosomes and the male X chromosome. The median expression value of the 11,952 autosomal genes was plotted against the median of the 371 X chromosome genes for 105 unrelated male individuals from four populations (Figure 1A). There is a clear linear relationship between autosomal and X gene expression in all populations. The majority of data points fall along a diagonal close to that where the autosomal median is equal to the X median. We compared mean expression of the 371 X genes with randomly selected sets of 371 autosome genes (*n* = 100) for 30 YRI males using Student's *t*-test and saw no significant difference (data not shown). Average expression from the single X chromosome in males is, therefore, similar to expression from an autosome pair.

Next, we compared the male X chromosome with each autosome pair separately. Results for the YRI population are shown in Figure 1B, and very similar results were obtained for the other populations (data not shown). Median expression from the single X chromosome falls within the normal range seen for autosome pairs and is slightly above average. The latter accounts for the observation that most data points in Figure 1A lie above the diagonal.

We conclude that expression from the single X in male cell lines is upregulated 2-fold relative to autosomes, thus achieving dosage parity between the X and autosomes. Upregulation occurs precisely and consistently in 105 individual male samples. This supports and extends the findings of Nguyen and Disteche [5].

### The Contribution of the Inactive X Chromosome in Females Is Small

The two X chromosomes in females are not equivalent as the majority of genes on one are subject to X inactivation; however, it is well established that a number of genes escape the silencing process. Therefore, we compared expression of X chromosome genes in females and males, reasoning that escape from XCI should produce a substantially higher level of expression in females.

**Figure 2:**
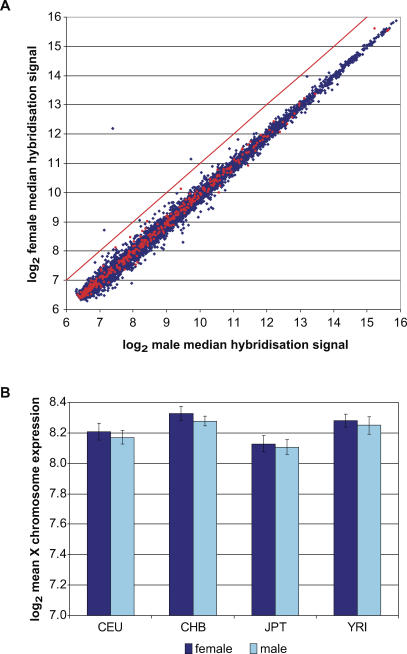
Limited Contribution of the X_i_ to Female Gene Expression (A) Median expression of 11,952 autosome genes (blue) and 371 X genes (red) for 30 YRI males plotted against median expression for 30 females. The red diagonal indicates the expected position of X-linked genes if expression were proportional to copy number. (B) Expression data from 371 X chromosome genes in 105 males and 105 females were normalised to the median of 11,952 autosomal sequences. The mean expression value for males and females in each population is illustrated. Error bars represent +/− 1 standard deviation.

First we compared expression of the 11,952 expressed autosome genes and 371 expressed X genes in 30 males and 30 females from the YRI population. Figure 2A shows median male expression plotted against female expression for each gene. The autosome genes lie on a diagonal with the vast majority showing very similar expression in males and females. Most X genes lie on the same diagonal, indicating that X chromosome gene expression too is similar in males and females and is not proportional to the number of X chromosomes. These data suggest that for most X chromosome genes, dosage compensation is achieved between males and females.

We then normalised expression of the 371 X chromosome genes to the median of the 11,952 autosome genes for each individual, and calculated the mean of the normalised X chromosome genes for 105 males and 105 females. In each population mean expression of X chromosome genes was higher in females than males (Figure 2B). However, these differences are small, representing increased expression in females of just 2.6%, 3.4%, 1.5%, and 2.2% for CEU, CHB, JPT and YRI, respectively. Genes escaping X inactivation do not, therefore, have a great effect on the overall level of X-linked gene expression in the female cell lines. This indicates that dosage compensation is occurring effectively.

### Identification and Analysis of Genes with Significantly Higher Female Expression

Although the difference is small, there is a measurable and consistent increase in X chromosome gene expression in females compared to males. In order to identify the genes that contribute to this difference, we looked for X chromosome genes with significantly higher expression in females. We used a Student's *t*-test to assess differences in expression levels between females and males within each population separately for the 371 X chromosome genes and 11,952 autosome genes (Table S1).

**Figure 3:**
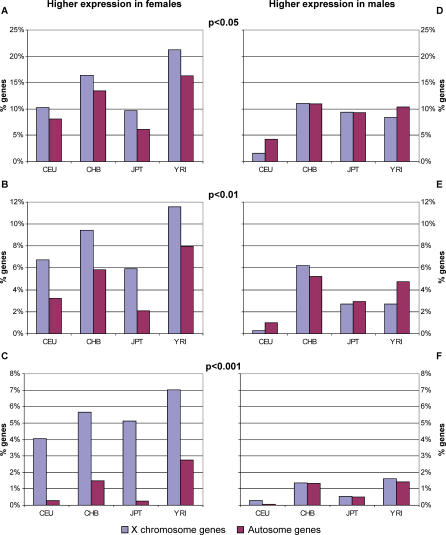
The Proportion of Genes with Higher Female Expression Is Greater from X Than Autosomes Percentage of X chromosome and autosome genes showing higher expression in females (A–C) or males (D–F) at three levels of significance: *p* < 0.05, *p* < 0.01, and *p* < 0.001. Note that the *y*-axis scale changes for different levels of significance.

Figure 3 compares the proportion of X chromosome and autosome genes expressed more highly in females or males at three different levels of significance: *p* < 0.05, *p* < 0.01 and *p* < 0.001. The proportion of X chromosome genes expressed more highly in females is greater than that from autosomes in each population. The difference between X and autosomes is consistent across the three levels of significance and at *p* < 0.001 ranges from 3.5% to 4.9% of genes in different populations. In contrast, the proportion of X chromosome genes with higher expression in males remains similar to that of autosome genes at all levels of significance (Figure 3).

The most likely explanation for the observations above is that a proportion of X chromosome genes escape from X inactivation to a measurable level in these female cell lines. We supposed that X-linked genes able to escape the silencing process would be expressed more highly in females in all populations. We also reasoned that other X-linked or autosomal genes reaching a significance threshold may not do so in all populations. We therefore assessed the population commonality of autosomal and X chromosome genes with higher female expression at different levels of significance (Table 1). At *p* < 0.05 and *p* < 0.01, the proportion of genes with significantly higher female expression is almost identical for autosomes and the X chromosome. However, the distribution of genes among populations is strikingly different, with a far greater percentage of X chromosome genes achieving significance in all four populations. When the significance threshold is raised to *p* < 0.001, the proportion of genes retained is now lower for autosomes than for the X chromosome, and no autosome gene is common to three or four populations (Table 1). By contrast, approximately 3% of X chromosome genes are significantly elevated in the females of all populations at *p* < 0.001 (Table 1). A single gene (*CD99*) is expressed more highly in the males of all four populations (*p* < 0.001). This is the only notable difference between X and autosomes in respect of higher male expression (Table 1).

**Table 1:**
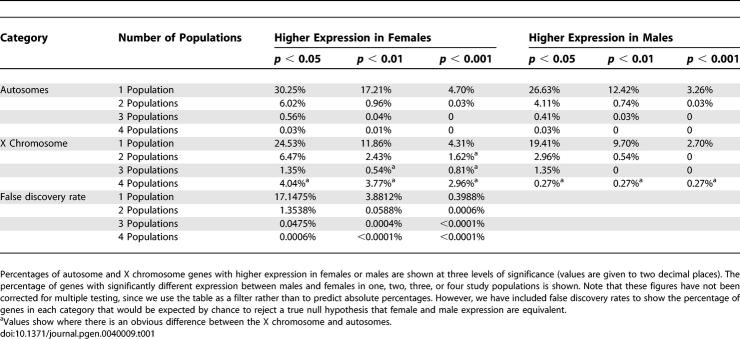
Observing a Clear Difference between X Chromosome and Autosome Genes in Respect of Higher Female Expression

Using the combination of significance values and population commonality as a filter (Table 1), we identified a group of 20 X chromosome genes that are remarkable in the consistency of their elevation across females (Table 2): *ALG13, CA5B, DDX3X, EIFIAX, EIF2S3, FUNDC1, HDHD1A, JARID1C, MSL3L1, PCTK1, PNPLA4, PRKX, RPS4X, SMC1L1, STS, UBE1, USP9X, UTX*, *ZFX* and *ZRSR2*. Eleven of these were expressed more highly in females at *p* < 0.001 in all four populations (Table 2). This situation was not observed for any of 11,952 autosomal genes tested.

**Table 2:**
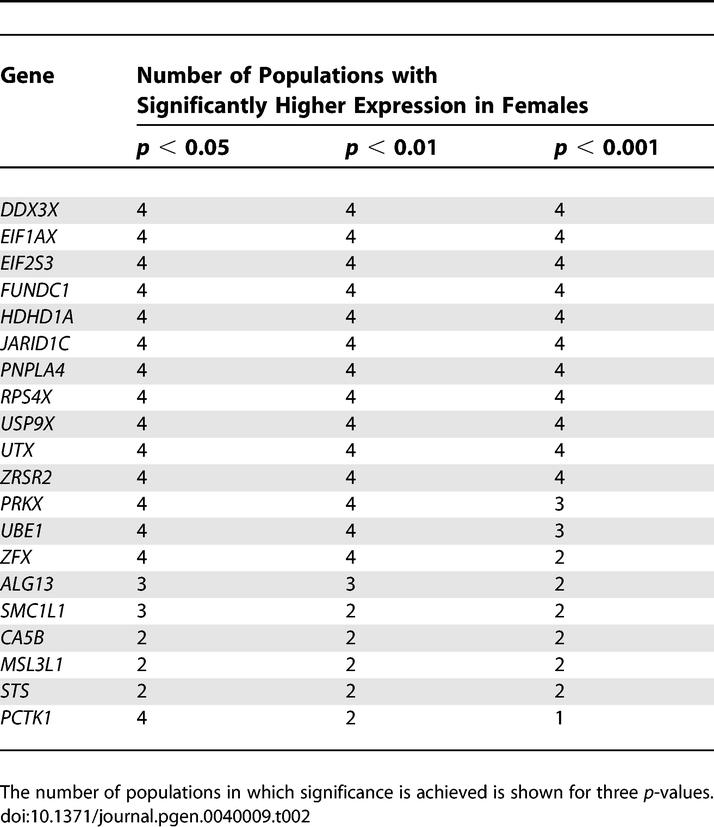
Genes Expressed More Highly in Females That Fit the Criteria for Escaping X Inactivation

**Figure 4:**
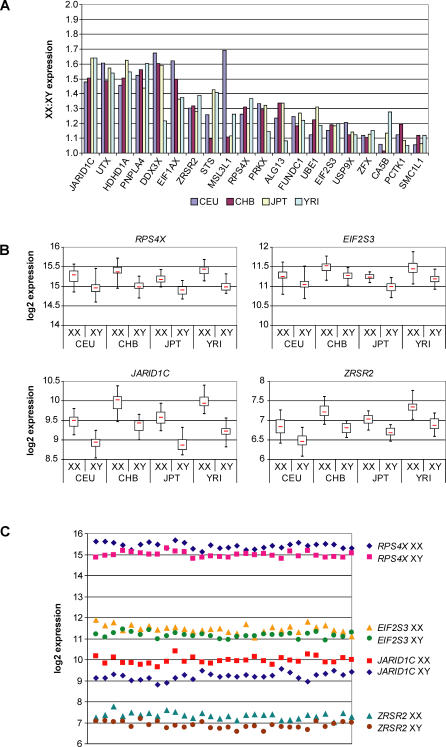
Comparison of Female and Male Expression of Genes with Higher Female Expression (A) Female to male expression ratio for 20 genes expressed more highly in females in four human populations. Expression in females is typically 10%–50% higher than in males. (B) Box and whisker plots showing the median, distribution, and range of expression for females and males for four X chromosome genes with higher female expression. The median is indicated by the red line, the box shows the interquartile range, and the ends of the whiskers the maxima and minima. The *y*-axis spans a 4-fold range for each graph to enable direct comparison of different genes. (C) Scatter plot of expression values of four non-dosage compensated genes in 30 males and 30 females from the YRI population. *JARID1C* is unique as none of the males has higher expression than any of the females. For *EIF2S3* the overlap is considerable, as only half the females have higher expression than the highest male and only five males express at a level below the lowest female.

Figure 4A illustrates the female to male ratio of expression for each of the genes in the four populations. Taking the mean of the four populations, there is a subset of six genes (*JARID1C*, *UTX*, *HDHD1A*, *PNPLA4*, *DDX3X* and *EIF1AX*) for which expression in females is around 1.5-fold greater than in males. Most genes have a much smaller difference: *EIF2S3*, *USP9X*, *CA5B* and *PCTK1*, *ZFX* and *SMC1L1* all have less than 1.2-fold higher expression in females compared to males. We interpret these ratios as expression from the active X that is equivalent to expression from the single X in males, combined with a lower level of expression from the inactive X that is more variable between genes. Some genes (e.g., *DDX3X*, *STS*) have a much higher ratio in some populations than others, which may represent a biological difference in the extent of escape from X inactivation in different human populations.

We observe higher female expression for 5.4% of the X chromosome genes expressed in the cell lines (20/371). The possibility remains that other X-linked genes may escape from XCI in lymphoblastoid cells. However, we have determined that these 20 genes account for almost all of the difference in gene expression between males and females seen in Figure 2B (data not shown). Therefore, any expression of additional genes from the inactive X chromosome must be very low and/or must occur in only a small fraction of female cell lines. In either case, the impact on dosage compensation at the population level would be minimal. Therefore, we conclude that 94.6% of X-linked genes are effectively dosage compensated in human lymphoblastoid cell lines.

### Genes with Higher Female Expression Do Not Show Greater Variation in Expression among Females

We hypothesized that escape from XCI may be partly stochastic, and that this might lead to greater variation in expression levels among females than males for some genes. Figure 4B shows box and whisker plots for four of the genes that can be considered to escape from XCI on the basis of their higher female expression. The other 16 genes follow a similar pattern (data not shown). The size of the box (interquartile range) is a good indicator of the similarity of distribution between females and males. In the majority of cases there is little difference in the distribution or range of values between the sexes, although the female values have a higher median and therefore the entire plot is shifted upwards. There is no correlation between the size of the interquartile range and median expression level. We also calculated the variance for each gene and found no significant difference between males and females within each population (unpublished data). We conclude that genes escaping XCI in these cell lines are not expressed more variably in females than males, which suggests that escape may be a tightly regulated rather than a stochastic event.

While the medians and interquartile ranges are clearly different between males and females, there is considerable overlap between the distributions of the expression levels of the two datasets. Individual data points from males and females of the YRI population are shown for four genes escaping XCI as a scatter graph (Figure 4C). *JARID1C* is unique in that all data points for females are higher than all data points for males. For the other 19 genes, the female and male datasets overlap to varying extents (contrast *EIF2S3* with *RPS4X* in Figure 4C). This can also be seen as overlap of whiskers in Figure 4B. This observation illustrates the extent of inter-individual variability of gene expression and highlights the importance of comparing large samples of males and females to identify differences in gene expression that are a consequence of escape from XCI.

### PAR1 Genes Have Equal Expression from X and Y Chromosomes

The genes from the pseudoautosomal regions (PARs) are a special case as they lie within regions of XY recombination and are essentially equivalent on the X and Y chromosomes. For genes in PAR1, escape from XCI is generally believed to be a prerequisite for dosage compensation between the two female X chromosomes and the male X and Y. We found that twelve PAR1 genes show no significant difference between females and males across populations and are therefore dosage compensated (Table S1). The only exception is *CD99*, which is expressed significantly more highly in males in all four populations (*p* < 0.001).

We identified single nucleotide polymorphisms (SNPs) in PAR1 genes *SLC25A6*, *CXYorf3*, *ZBED1* and *CD99* and used a quantitative assay to measure relative expression from the X and Y alleles in heterozygous males. As shown in Table 3, the relative contribution from the X and Y chromosomes is very similar for each of the four genes. We conclude that the majority of PAR1 genes escape from XCI and are dosage compensated, consistent with the expectation above.

**Table 3:**
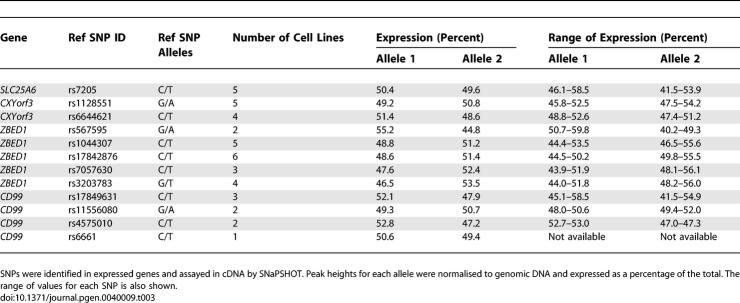
Genes in the Pseudoautosomal Region (PAR1) Are Expressed Equally from the X and Y Chromosomes in Male Cell Lines

### Evidence That Dosage Compensation of Some X-Linked Genes Is Maintained through Expression of a Y-Linked Copy

**Figure 5:**
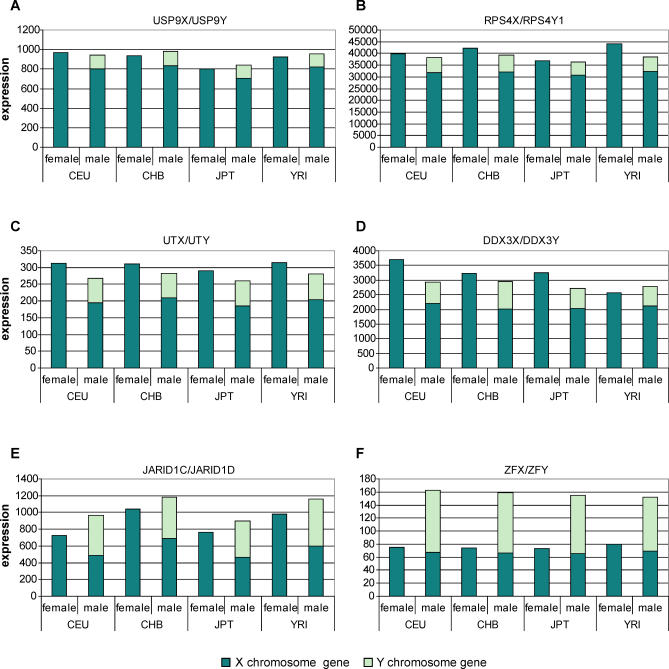
Evidence That Expression of the Y-linked Copy of XY Gene Pairs Can Maintain Dosage Compensation (A–F) Stacked bars show the cumulative expression of X and Y gametologues in males alongside expression from the X chromosomes in females. Note that the *y*-axis is a linear scale.

The majority of genes that have a functional Y chromosome homologue were found to be expressed in hybrid cells containing the X_i_ [16]. Therefore, we decided to test the possibility that genes with higher female expression might, like the genes in PAR1, be compensated by functionally equivalent Y-linked copies. Eight of the 20 genes with higher female expression have functional Y-linked gametologues. We excluded *PRKY* and *EIF1AY* from the analysis. The *PRKY* probe has 94% sequence identity to *PRKX* and gives a strong signal in females. Expression of *EIF1AY* is approximately 13-fold greater than *EIF1AX* in males, suggesting that *EIF1AY* is not involved in a compensation mechanism. Gene expression for the remaining six X-Y gene pairs is shown in Figure 5.

*USP9X* expression is significantly higher in females at *p* < 0.001 in all populations. Interestingly, the sum of *USP9X* and *USP9Y* expression in males is not significantly different from the level of *USP9X* in females (*p* = 0.822 [CEU], *p* = 0.024 [CHB], *p* = 0.245 [JPT] and *p* = 0.610 [YRI]). *USP9Y* expression therefore completely restores the *USP9X* dosage imbalance between males and females. Figure 5B–5D shows similar results for gene pairs *RPS4X*/*RPS4Y1*, *UTX*/*UTY* and *DDX3X*/*DDX3Y*. In each case dosage from the X and Y copies in males is similar to that of the X copies in females. Expression of the Y copy is always much lower than expression of the X copy and appears to reflect the expression from the X_i_. For these four genes, dosage compensation appears to be achieved by expression of the Y copy. In contrast, expression levels of *JARID1C* (X) and *JARID1D* (Y) (Figure 5E) are approximately equal and their combined expression in males is significantly greater than *JARID1C* expression in females. A similar picture is obtained with *ZFX* and *ZFY* in males (Figure 5F). We have identified an X-Y gene pair (*TMSB4X*/*TMSB4Y*) whose X copy is dosage compensated according to our data. *TMSB4Y* is expressed at less than 1% of the level of *TMSB4X* and therefore does not affect dosage compensation between males and females.

### Genes with Higher Female Expression Are Not Distributed Evenly on the X Chromosome

**Figure 6:**
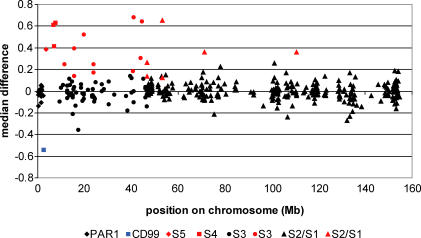
Genes Expressed More Highly in Females Are Distributed Non-Randomly on the X Chromosome For each gene, the median expression value for males was subtracted from the median expression value for females to give a median difference. Illustrated is the median difference for each gene based on averaging the values for the four populations, plotted according to location on the X chromosome. A median difference of 1 represents a 2-fold difference in expression. Different symbols represent different evolutionary strata. Genes expressed significantly more highly in females are coloured red, and the single gene expressed significantly more highly in males (*CD99*) is coloured blue.

Genes that escape from XCI in hybrid cell lines are non-randomly distributed on the X chromosome [16,17]. In light of identifying a smaller proportion of genes with higher female expression, we assessed the relationship between gene expression and chromosomal location. We observed that the distribution of genes with elevated female expression is also non-random, with most lying on the short arm (Figure 6). The chromosome can be divided into strata that ceased to recombine with the Y chromosome at different times in evolutionary history [17,26]. The most ancient parts of the chromosome (strata S1 and S2), covering the long arm and proximal short arm, contain 287 of the 371 expressed genes, but only six that have higher female expression. By contrast, a larger fraction of genes have elevated female expression levels in regions that stopped recombining with the Y chromosome more recently, either in early eutherian mammals (S3) or in primates (S4, S5). Ten out of 66 S3 genes fit this picture, while all three expressed genes in S4, together with the single example in S5, are more highly expressed in females. These findings support the model that X-linked genes are recruited into the XCI system following the Y chromosome degeneration that occurs when regions cease to recombine [27].

## Discussion

On the basis of these data, we suggest that dosage compensation in human lymphoblastoid cells is virtually complete. Gene expression from the single X chromosome in males is upregulated 2-fold compared to the autosomes. Expression from the female X chromosome pair is almost the same as from the male X, suggesting that few genes escape the silencing process to any great extent. Twenty genes in this study (5.4%) have significantly higher female expression, and four of these could have dosage balance maintained through expression of a Y-linked homologue.

Ohno predicted 40 years ago that the evolution of a dosage compensation mechanism in mammals must have involved a doubling of expression of each X-linked gene as the Y chromosome degenerated [4]. Two-fold upregulation of the X chromosome has now been demonstrated for both humans and mice [5,6] and in a range of tissues [5]. In D. melanogaster, a slight but significant overexpression of the X chromosome compared to the autosomes in all XX;AA samples has been reported [6] which may be due to inherent hypertranscription of the X chromosome. We have determined that human X chromosome expression is not significantly elevated above the autosome average, suggesting that the X chromosome is not hypertranscribed in the cell lines over and above the 2-fold upregulation. Our data show that upregulation occurs precisely and consistently in 105 individual male samples, also leading us to conclude that in lymphoblastoid cell lines gene expression is appropriately regulated.

Upregulation of the X is not seen in mouse germ cells suggesting that it takes place in the developing embryo [5]. Two-fold upregulation may be a general feature of X chromosomes, affecting genes on X_a_ and those on X_i_ that escape inactivation. Alternatively, upregulation and silencing could be mutually exclusive choices for X chromosomes in embryogenesis, simultaneously achieving correct X gene expression and dosage compensation. Previously, genes have been classified as partially escaping XCI if their female to male expression ratio is below two. However, under the second model described, genes fully expressed from both X_i_ and an upregulated X_a_ in females would have a theoretical maximum expression that is 1.5-fold greater in females than males. We favour this model as we have identified six genes expressed approximately 1.5-fold more highly in females and the greatest ratio we observe for any gene is 1.56, averaged across four populations.

Is 2-fold upregulation a chromosome-wide phenomenon? The PAR1 region is the only surviving remnant of a large autosomal addition to both sex chromosomes that still undergoes recombination in male meiosis. The X and Y chromosomes are equivalent in PAR1 and genes here are predicted to escape XCI. Accordingly, all PAR1 genes tested were expressed from X_i_ in hybrid cell lines [16]. The majority of PAR1 genes included in our study showed no significant difference in expression between females and males. This gives rise to two models for PAR1 gene expression. In the first, PAR1 genes have equal expression from X_a_, X_i_ and Y copies and so no dosage compensation is necessary. Under this model, PAR1 is excluded from both the upregulation and the silencing components of dosage compensation. In the second model, the PAR1 is 2-fold upregulated on the X_a_ only and dosage compensation is achieved through equal expression from the X_i_ and Y copies. A corollary of this model is unequal expression of PAR1 alleles within a cell, but equal expression between males and females. We have tested three dosage compensated PAR1 genes in males and find that expression levels are similar from the X and Y alleles. Therefore, we favour the first model and propose that PAR1 is protected from both upregulation and silencing.

*CD99* is the only PAR1 gene found to be more highly expressed in males, yet has equivalent expression from the X and Y copies. *CD99* is the gene that lies closest to the boundary between the PAR1 and X-linked material, and we suggest that spreading of the XCI signal across the pseudoautosomal boundary results in partial silencing of *CD99*. Consistent with this hypothesis, the protein product of the *CD99* gene was found to be present at lower levels in hybrids containing X_i_ than those containing X_a_ [28].

Outside the pseudoautosomal regions, twenty genes are expressed at significantly higher levels in female cell lines. We hypothesized that these genes would escape from XCI. Formally, the alternative explanation for the elevated female expression could be that these genes are hypertranscribed from the female active X chromosome. However, since all of these genes are included in previous reports of escape from XCI [12,15,16,29–33], we prefer the former explanation for higher female expression. An intriguing observation is that the dosage of some of these genes could be effectively compensated by expression of a Y copy. An underlying assumption of this analysis is that the X and Y gametologues are functionally equivalent, despite their evolutionary divergence. The DEAD box RNA helicase proteins DDX3X and DDX3Y appear to be interchangeable, as both rescue a temperature-sensitive mutant hamster cell line incapable of growth at a non-permissive temperature [34]. The ribosomal proteins RPS4Y1 and RPS4X show functional equivalence in a similar rescue assay and can function interchangeably in ribosomes [35]. *RPS4Y1* and *RPS4X* are among a very small number of genes from the ancient sex chromosomes that have functional Y copies [17]. Despite their considerable divergence time and very high synonymous substitution rate, their protein products share 93% identity and are the same length, consistent with their being functionally equivalent.

Previous microarray studies have assessed escape from XCI by looking for increased expression in cell lines with supernumerary X chromosomes [18] or by comparing female and male expression [19,20]. More recently, a larger study assessed genome-wide sex differences in gene expression using lymphoblastoid cell lines from monozygotic twin pairs [21]. Each study reports some genes that are considered to be well established as escaping from XCI, but the four vary considerably in the number and identity of genes documented. These differences might be explained in part by variation in escape from X inactivation in different tissues, for which there is evidence [20]. However, a further possibility is that inter-individual variability in expression that is unrelated to XCI could increase the risk of false positives or negatives where the sample size is small. The scale of our study, which measured gene expression in 210 individuals for 81% of protein coding genes on the X chromosome, means that we can confidently detect significantly higher expression in females at the population level in spite of this factor. Notably the study by McRae *et al*. [21], which assayed 38 lymphoblastoid cell lines, agrees most closely with our study in the identity and proportion of genes with higher female expression.

We find that 5.4% of X-linked genes have increased female expression in the cell lines. Analysis of somatic-cell hybrids that retain X_i_ and of fibroblast cells with non-random XCI has put the proportion of genes escaping XCI at 15%–25% [16]. This difference could be explained by a lower proportion of genes escaping XCI in lymphoblastoid compared with fibroblast (or hybrid) cells. However, perhaps more important are the differences of approach between the two studies. Our study used a population based analysis of dosage compensation, whereas Carrel and Willard [16] detected expression in hybrid cell lines, or compared expression levels from X_a_ and X_i_ alleles in female cell lines, using methods capable of detecting very low levels of expression from X_i_. Some genes, therefore, could be expressed from X_i_ but at a level that is insufficient to cause a dosage imbalance. Other genes could escape to a larger extent in a small number of females, as suggested by Carrel and Willard [16]. Neither of these, though, is substantial enough to generate a significant sex-difference at the population level.

We conclude that dosage compensation in human lymphoblastoid cell lines is highly effective and tightly controlled. It will be interesting to extend these studies to other tissues, but it seems unlikely that this level of regulation would be restricted to this single cell type. Therefore, we propose that the contribution of escape from XCI to male-female phenotypic differences may be small and furthermore, we suggest that the number of genes contributing to phenotype in X chromosome aneuploidies is lower than previously thought.

## Materials and Methods

### Generation of dataset.

Gene expression was assayed in lymphoblastoid cell lines of all 210 unrelated HapMap individuals [25] from four populations (CEU: 60 (30 Male/30 Female) Utah residents with ancestry from northern and western Europe; CHB: 45 (22M/23F) Han Chinese in Beijing; JPT: 45 (23M/22F) Japanese in Tokyo and YRI: 60 (30M/30F) Yoruba in Ibadan, Nigeria). RNA preparation, labeling, hybridization to Sentrix Human-6 BeadChip (Illumina), gene expression quantification and normalization of raw data were described previously [23]. Briefly, each RNA sample was labelled in duplicate and each labelled sample was hybridised to two separate arrays. Data were subjected to quantile normalization then were median normalized across all individuals. Final data points for each gene are the mean of the four normalized hybridisation values. Log_2_ transformed mRNA expression values were used throughout except where otherwise stated. Data can be downloaded from http://www.sanger.ac.uk/humgen/genevar/ and the Gene Expression Omnibus (http://www.ncbi.nlm.nih.gov/geo/) entry GSE6536.

### Selection of expression data for analysis.

We established an appropriate cut-off point for evaluating gene expression by examination of four non-human probes (lysA, pheA, thrB and trpF) in 210 individuals: 836/840 data points had log_2_ expression values <6.4. We also evaluated signals from Y-linked genes in female samples: 593/620 data points were found to have values <6.4, excluding two probes that apparently cross-hybridised with X-linked genes. We conservatively chose to analyse genes with log_2_ median expression >6.4 in all four populations. We excluded redundant probes (*n* = 21) for X chromosome genes and any X probes that matched autosomal exons. Data for 11,952 expressed autosome sequences and 371 expressed X chromosome genes were used in all downstream analyses. The complete set of X chromosome and autosomal genes represented in this study and median expression values are shown in Table S2.

### Statistical analysis.

Data from the four populations were considered separately throughout. We tested male and female datasets separately for skewness and kurtosis for 371 X chromosome probes and found no evidence for them, except for a very small number of genes in some populations where log_2_ expression was close to the expression cutoff of 6.4. We therefore concluded that the gene expression data generally follow a normal distribution. We used median values to illustrate chromosome or population averages except where we have shown the standard deviation (Figure 2B). To make comparisons between autosome genes and X genes, or between groups of individuals, we normalized all gene expression values to the median value of 11,952 autosomal probes for each individual. We compared the variances of male and female samples for X chromosome genes by placing the larger variance over the smaller to form an F statistic. We found that variances for females and males are not significantly different. We tested significance by calculating *p*-values associated with a Student's two sample homoscedastic *t*-test with a two-tailed distribution. The complete list of *p*-values is shown in Table S1.

### Allele-specific gene expression analysis.

SNaPshot was carried out on cDNA and genomic DNA from heterozygous males using the SNaPshot Multiplex Kit (Applied Biosystems) according to the manufacturer's instructions with the following modifications. Initial template generation was carried out using Platinum Taq polymerase (Invitrogen) in a standard reaction using touchdown polymerase chain reaction (PCR): denaturation: 94°C 15 min; 20 cycles: 94°C 30 sec, 70°C, 30 sec reducing by 1°C per cycle, 72°C 45 sec; then 15 cycles: 94°C 30 sec, 50°C 30 sec, 72°C 45 sec; final extension 72°C for 7 min. PCR products were treated with 2 units of shrimp alkaline phosphatase (USB) and 1.5 units of Exonuclease I (USB) for 1 hour at 37°C to remove primers and nucleotides, then at 80°C for 15 mins. Primer extension products were analysed on an ABI 3730 DNA Analyzer with a POP-7 Polymer and a 36cm capillary array with the ABI standard run module. SNP data were analysed using ABI PRISM GeneMapper Software Version 3.0. Primers used to generate template DNA for analysis and SNaPshot extension primers are shown in Table S3. Peak heights from cDNA were normalized to genomic DNA values and expressed as an allelic ratio.

## Supporting Information

Table S1 XX and XY Normalised Medians and Means and *p*-Value Associated with Student's *t*-test (5.5 MB XLS)

Table S2 Population Median Values for 11,952 Autosome and 371 X Genes (2.0 MB XLS)

Table S3 SNaPshot Primers (5′–3′) (21 KB XLS)

## Abbreviations

PAR1: pseudoautosomal region 1
XCI: X chromosome inactivation
X_a_: active X chromosome
X_i_: inactive X chromosome
